# Silhouette-Length-Scaled Gait Parameters for Motor Functional Analysis in Mice and Rats

**DOI:** 10.1523/ENEURO.0100-19.2019

**Published:** 2019-10-30

**Authors:** Ivanna K. Timotius, Sandra Moceri, Anne-Christine Plank, Johanna Habermeyer, Fabio Canneva, Jürgen Winkler, Jochen Klucken, Nicolas Casadei, Olaf Riess, Bjoern Eskofier, Stephan von Hörsten

**Affiliations:** 1 Machine Learning and Data Analytics Lab, Department of Computer Science, Faculty of Engineering, Friedrich-Alexander-University Erlangen-Nürnberg (FAU), 91052 Erlangen-Nürnberg, Germany; 2 Department Experimental Therapy, University Hospital Erlangen (UKEr) and Preclinical Experimental Animal Center, Friedrich-Alexander-University Erlangen-Nürnberg (FAU), 91054 Erlangen-Nürnberg, Germany; 3 Department of Molecular Neurology, University Hospital Erlangen (UKEr), Friedrich-Alexander-University Erlangen-Nürnberg (FAU), 91054 Erlangen-Nürnberg, Germany; 4 Department of Electronics Engineering, Satya Wacana Christian University, Salatiga 50711, Indonesia; 5 Institute of Medical Genetics and Applied Genomics, University of Tübingen, 72076 Tübingen, Germany

**Keywords:** CatWalk, gait, Huntington, normalization, Parkinson, scaling

## Abstract

Gait analysis of transgenic mice and rats modeling human diseases often suffers from the condition that those models exhibit genotype-driven differences in body size, weight, and length. Thus, we hypothesized that scaling by the silhouette length improves the reliability of gait analysis allowing normalization for individual body size differences. Here, we computed video-derived silhouette length and area parameters from a standard markerless gait analysis system using image-processing techniques. By using length- and area-derived data along with body weight and age, we systematically scaled individual gait parameters. We compared these different scaling approaches and report here that normalization for silhouette length improves the validity and reliability of gait analysis in general. The application of this silhouette length scaling to transgenic Huntington disease mice and Parkinson´s disease rats identifies the remaining differences reflecting more reliable, body length-independent motor functional differences. Overall, this emphasizes the need for silhouette-length-based intra-assay scaling as an improved standard approach in rodent gait analysis.

## Visual Abstract

**Figure:**
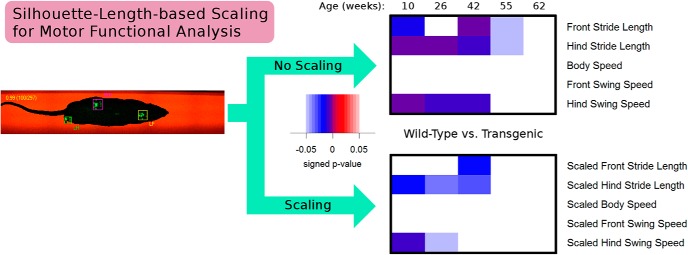

## Significance Statement

Body size corresponds to gait parameters in both human and rodents. Using image-processing approaches, we computed the silhouette length of the rodent and demonstrated that it is correlated to the stride length, body speed, and swing speed. Subsequently, as a proof of concept, we used the resulting silhouette length for the scaling/normalization of these gait parameters of two rodent models for neurodegenerative disorders, namely Parkinson’s disease-relevant rat model and Huntington disease-relevant mouse model. Genotype-related alterations for silhouette length, stride length, body speed, and swing speed became smaller by applying our body silhouette-length-based scaling. In general, this silhouette-length-based gait parameter scaling is necessary for gait analysis in rodents, especially in studies involving young rodents, in preclinical longitudinal studies, and in studies with genotype-related body size difference.

## Introduction

Motor function analysis is essential for studies of disorders characterized by motor impairments, such as Parkinson’s disease (PD; Tolosa et al., 2006; Jankovic, 2008) and Huntington disease (HD; Roos, 2010). A video-based markerless gait analysis tool for rodents, namely the CatWalk XT system (Hamers et al., 2001, 2006; Noldus Information Technology b.v., 2012), has been used in some preclinical studies. The CatWalk system quantifies several gait parameters based on the footprints and the body silhouettes of animals walking on an illuminated walkway in a darkened room. To continuously improve the outcome of the CatWalk-derived data acquisition, we recently described methods for the characterization of swaying movement in PD-relevant mouse models (Timotius et al., 2018b,c).

Recent publications reveal the association of several factors on gait parameters, such as body size (Heglund et al., 1974; Taylor, 1978; Machado et al., 2015), sex (Datto et al., 2016), strain, and locomotion speed (Taylor, 1978; Webb et al., 2003; Koopmans et al., 2007; Neckel et al., 2013; Machado et al., 2015; Neckel, 2015). Although the body size of rodents might influence gait analysis, the scaling of these parameters is not yet common in rodent gait analysis. Most researchers use adult rodents, where the body size is relatively similar and stable, and/or compare their preclinical models with wild-type controls of the same age (Cao et al., 2008; Vandeputte et al., 2010; Wang et al., 2012; Casadei et al., 2014; Chen et al., 2014; Ishikawa et al., 2014; Jacobs et al., 2014; Salvi et al., 2014). However, such an approach will not correct for genotype-driven body size differences as characteristic of some transgenic (TG) rodent models of human disorders including but not limited to HD (von Hörsten et al., 2003; Clemensson et al., 2017) and PD (Kohl et al., 2016). Body size-related gait parameter scaling appeared in a very few research studies (e.g., body-weight-based scaling of stride length measured by CatWalk; Frahm et al., 2018 and body-weight-based scaling of gait parameters measured by LocoMouse; Machado et al., 2015). For the reason that in the human gait analysis, body-height- or leg-length-based scaling is recommended for several gait parameters (Hof, 1996), we hypothesized here that body length-based scaling is more reliable for gait parameter scaling of rodents compared with body-weight-based scaling.

Body length (nose-to-anus length or nose-to-tail-base length) calculation in rodents was applied in several research works, in most cases of which, however, for this purpose anesthesia had to be applied to standardize and to keep the rodent motionless (Novelli et al., 2007; Dunn and Bale, 2009; Chakraborty et al., 2017). Such anesthesia procedures might add a confounding factor to the study design of the experiment (Karwacki et al., 2001) and, therefore, if possible, should be avoided. Therefore, direct *in vivo* measurements of body-length-related parameters during the execution of gait testing would offer the advantage of recording both body-length-related measurements in a functional fashion as well as gait-specific parameters in the same run, enabling longitudinal normalization of data collected from rodents from a young age until advanced adulthood, as often desired while investigating animal models of progressive degenerative conditions.

Body-length-related parameter computation method in a with-marker gait analysis system cannot directly be applied to a markerless gait analysis system. As a markerless video-based gait analysis tool, the CatWalk system (Hamers et al., 2001, 2006; Noldus Information Technology b.v., 2012) provides silhouette information along with the gait parameters. The silhouette length is directly related to the body length of the rodent. Therefore, CatWalk gait parameter scaling can be performed based on the silhouette length. This silhouette-length-based scaling connects the footprint-based gait parameters with the recorded body silhouettes of the walking animal. Therefore, we here considered the following aims:

1. To propose silhouette length and area computation methods based on image-processing techniques.
2. To investigate the correlation between silhouette length and several gait parameters.
3. To compare silhouette length and measure body length.
4. To propose a gait parameter scaling method based on the silhouette length.
5. To compare the silhouette-length-based scaling with the silhouette-area-, body-weight-, and age-based scaling.
6. To apply these methods to a mouse model and a rat model of neurodegenerative disorders exemplifying two species models of disease and allowing more reliable identification of genotype-driven differences.

Here we report, that, even in wild-type mice and rats, the computed body silhouette length is correlated with the stride lengths and speed-related parameters, thus describing that body silhouette length and motor function are interconnected. In fact, silhouette-length-based scaling lowers this correlation. We deliver supportive data that this silhouette-length-based scaling on the stride lengths and speed-related parameters cannot be replaced by other normalization factors such as the silhouette area, body weight, or age. Finally, we demonstrate that gait parameter differences between genotypes across age are more reliably detected by the application of our scaling approach using silhouette length differences.

## Materials and Methods

### Subjects

**Figure 1.**
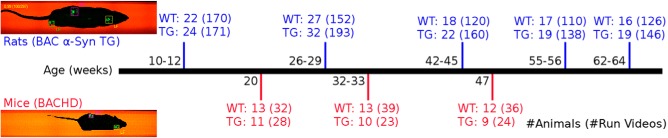
Scheme of experimental design. Depicted images were captured from the CatWalk video recordings providing information about the body silhouette and paw positions. The timeline displays the number of rodents and run videos analyzed within a longitudinal, repeated experimental design spanning five age points for rats and three age points for mice.

The animals used for this study were male bacterial artificial chromosome (BAC) α-synuclein (α-Syn) TG rats (Nuber et al., 2013; Kohl et al., 2016; Moceri et al., 2018) as a PD-relevant TG model, and BAC of mutant *HTT* gene (BACHD) mice (Gray et al., 2008), backcrossed to C57BL/6N, as an HD-relevant transgenic model and their corresponding wild-type littermates. The numbers of animals involved in the study are given in Figure 1. Parts of the data were presented at the Measuring Behavior Conference 2018, Manchester, U.K. (Timotius et al., 2018d). Data acquisition for the BAC α-Syn transgenic rats was presented at the AD/PD Meeting 2018 (Moceri et al., 2018). Additionally, for the purpose of comparing the video-based measured silhouette length and the corresponding manually measured body length, 10 additional BAC α-Syn rats were used in this study.

The rodents were maintained under specific pathogen-free conditions with a 12 h light/dark cycle and food and water available *ad libitum*. All research and animal care procedures were performed in compliance with international animal welfare standards and approved by the district of Lower Franconia, Würzburg, Bavaria, Germany (RegUFr#55.2-2532-2-218/54-2532.1-49/12).

### Study protocol and data acquisition

Data acquisition was performed using the CatWalk system (Hamers et al., 2001, 2006; Noldus Information Technology b.v., 2012), a markerless gait analysis tool equipped with an enclosed corridor on a glass floor. As an animal walks through the alley, its body silhouette and paw contact positions are recorded by a video camera positioned under the walkway at a sampling rate of 100 Hz. Figure 1 gives examples of images captured from the recorded video. Footprint positions are labeled automatically by the CatWalk software and revised manually by an experienced observer. Several gait parameters are then automatically calculated based on these labeled footprints.

During the data collection process, rodents were free to walk from one end to the opposite end of the alley with no need for physical restrictions or rewards. The walking speeds were 30.7 ± 6.7 cm/s for rats and 30.3 ± 7.7 cm/s for mice (mean ± SD). All runs were recorded in the dark at a minimum level of external disturbing factors. Data were acquired between their young and advanced age at five different age points for rats and three different age points for mice, over several acquisition days for each age point. The number of animals and runs for each age point is given in Figure 1. Intraindividual longitudinal data were collected from a subgroup of animals in each group, thus allowing further investigation of the effect of growth on the gait parameters. The minimum number of individual repeated measures across ages was 16 for rats and 9 for mice.

For the comparison of the different scaling methods, the body weight of wild-type rodents was measured at ±1 week from the CatWalk gait data acquisition. The number of rats with body weight information was 22, 27, 17, and 16 assessed at the ages of 10–12, 26–29, 55–56, and 62–63 weeks, respectively. In addition, the number of mice with body weight information was 13, 13, and 12 assessed at the age of 21, 32, and 46 weeks, respectively.

Manual measurement of the body length for the additional 10 BAC-α-Syn rats was performed on isoflurane anesthetized rats by a physically measurement of the nose to tail-base length. All measurements have been performed on the consecutive day after the recording of the CatWalk video files for in total 67 runs of these animals.

Based on human motor functional analysis studies (Hof, 1996), human gait parameters related to length, duration, frequency, speed/velocity, moment, energy, power, angular velocity, angular acceleration, as well as the moment of inertia should be scaled by the human leg length or height. Not all of these gait parameters are provided in the CatWalk rodent gait analysis system. Therefore, the following parameters, *p_g_*, were chosen from the CatWalk-derived parameters for this study:

1. Stride lengths:
  (a) Front-paw stride length (cm)
  (b) Hind-paw stride length (cm)
2. Parameters relating to duration:
  (a) Front-paw stand time (s)
  (b) Front-paw swing time (s)
  (c) Front-paw step cycle (s)
  (d) Hind-paw stand time (s)
  (e) Hind-paw swing time (s)
  (f) Hind-paw step cycle (s)
3. Speed parameters:
  (a) Body speed (cm/s)
  (b) Front-paw swing speed (cm/s)
  (c) Hind-paw swing speed (cm/s)

The parameters obtained from the left and right paws were averaged, with the exception of body speed, which was averaged over all four paws.

### Silhouette length and silhouette area computation

**Figure 2.**
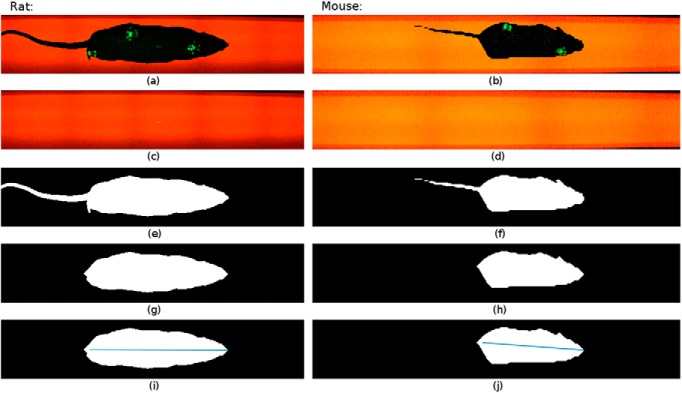
Scheme of image-flow analysis. ***a–j***, Images captured from the unlabeled CatWalk videos (***a***, ***b***); background images (***c***, ***d***); background subtraction results *I*_FG_ (***e***, ***f***); binary largest objects *I_R_* (***g***, ***h***); silhouette length illustration (***i***, ***j***): rat with a silhouette length of 23.9 cm and mouse with a silhouette length of 9 cm.

The silhouette length and silhouette area were computed using Matlab R2015a (8.5.0.) based on the unlabeled video and its corresponding background image. Representative images are shown in Figure 2*a–d*. The silhouette length computation begins at the first image frame where all paws are detected on the walkway and ends at the image frame where the first paw disappears from the recording area. These start and end points are obtained based on the information given by the CatWalk run data. This run data provides information on the labeled paw positions in each frame. Given that *f_RH_*, *f_LH_*, *f_RF_*, and *f_LF_* are vectors containing the frame number in ascending order of video frames with detected right hind-paws, left hind-paws, right front-paws, and left front-paws, respectively, *f_RH_*(1) and *f_LH_*(1) refer to the first appearance of the corresponding paws and *f_RF_*(last) and *f_LF_*(last) refer to the last appearance of the corresponding paws. The start and end frames are therefore obtained as follows:

(1) $$f_{start}=\max\left\{f_{RH}\left(1\right),f_{LH}\left(1\right)\right\}$$

(2) $$f_{stop}=\min\left\{f_{RF}\left(last\right),f_{LF}\left(last\right)\right\}$$

**Figure 3.**
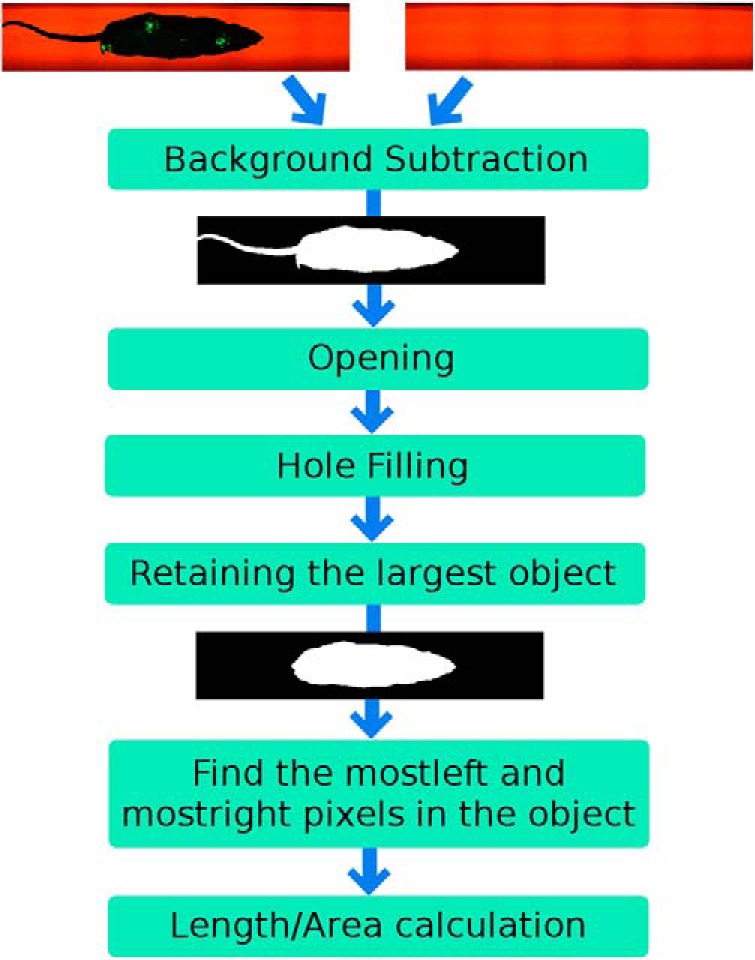
Block diagram of the silhouette extraction, tail removal, and silhouette parameters.

For each frame between the start (*f*_start_) and end (*f*_stop_) points, the silhouette extraction and tail removal are depicted in Figure 3 and are explained as follows: background subtraction is performed in order to extract the rodent’s body [foreground (*I*_FG_)] from the background (*I*_BG_). The foreground *I*_FG_ of each image frame *I_O_* is extracted by choosing the image pixels that have a pixel value difference more than a threshold *T_h_* = 10 in at least one of the color layers (*I_L,R_*, *I_L,G_*, or *I_L,B_*) compared with the background image *I*_BG_. The value of the threshold *T_h_* was chosen to avoid noise oversensitivity. Some examples of the resulting images after background subtraction are shown in Figure 2, *e* and *f*, as follows:

(3) $$I_{L}=\left|I_{O}-I_{BG}\right|>T_{h}$$

(4) $$I_{FG}=I_{L,R}\,\;OR\,\;I_{L,G}\,\;OR\;I_{L,B}$$

Morphological image opening (Gonzalez and Woods, 2008): removal of the tail part from the image is performed via morphological opening using a diamond structuring element (*I*_SE_). This diamond shape was chosen to best preserve the without tail body shape. The distance *r_d_* from the structuring element origin to the points of the diamond was adjusted to the tail size of the rodents, which are *r_d_* = 9 mm (corresponding to 9 pixels with image resolution, *x_mm_* and *y_mm_*, 1 mm/pixel) for rats and *r_d_* = 5 mm (corresponding to 7 pixels with image resolution, *x_mm_* and *y_mm_*, 0.7 mm/pixel) for mice.

Hole filling (Gonzalez and Woods, 2008): this operation fills any hole in the object of the image *I_T_*. The resulting image is denoted by *I_H_*.

Retaining the largest object: the largest object in the image is then the body silhouette. Examples from the resulting image *I_R_* are shown in Figure 2, *g* and *h*.

The length of the silhouette is then calculated by first detecting the positions of the leftmost and rightmost pixels of the body silhouette that is retained in *I_R_*, as follows: (*x*_1_, *y*_1_), (*x*_2_, *y*_2_). Then, the Euclidean distance between these two points is calculated as follows:

(5) $$l_{sil}=\sqrt{{\left\{\left(x_{1}-x_{2}-r_{d}\right)\cdot x_{mm}\right\}}^{2}+{\left\{\left(y_{1}-y_{2}\right)\cdot y_{mm}\right\}}^{2}}$$

The image resolution *x_mm_* and *y_mm_* is indicated by the exported CatWalk data. The subtraction of *r_d_* is performed since in most cases half of the structure element area *I*_SE_ belongs to the tail area. Illustrations of the length are shown in Figure 2, *i* and *j*, where the image resolutions for these specific videos are 1 mm/pixel for the rats and 0.7 mm/pixel for the mice.

The area of the silhouette is calculated by counting the number of pixels in the silhouette that is retained in *I_R_*, followed by multiplying it with the image resolution *x_mm_* and *y_mm_*. It is noteworthy that the silhouette area can be calculated from the silhouette (blob) including the tail, *I*_FG_, or excluding the tail, *I_R_*, according to the application need. After the silhouette length and area are calculated for each frame, the silhouette length and area of the whole run video are finally determined by the maximum length and area values from all the calculated lengths and areas of the video frames. By doing this, a value of silhouette length and a value of silhouette area are specified for every run video.

For all animals, where body weight information (*w*) is additionally available, a body weight silhouette length index, *I*_WSL_, can also be calculated. As silhouette length is related to body length, this index resembles the body mass index (Novelli et al., 2007), as follows:

(6) $$I_{WSL}=\frac{w}{l_{sil}^{2}}$$

### Gait parameters scaling method

The gait parameters *p_g_* are scaled by dividing their values by the silhouette length as indicated in Equation 7. This yields the silhouette-length-scaled gait parameters ${\hat{p}}_{g}$, as follows:

(7) $${\hat{p}}_{g}=\frac{p_{g}}{l_{sil}}$$

For the parameters related to speed, an additional scaling method is also tested. This additional scaling method is adapted from human gait analysis (Hof, 1996). It is given in Equation 8, where *g* is the acceleration of gravity (9.81 m/s^2^ on earth). This yields the silhouette-length-scaled gait parameters ${\hat{p}}_{a}$, as follows:

(8) $${\hat{p}}_{a}=\frac{p_{g}}{\sqrt{\frac{g}{l_{sil}}}}$$

For the comparison of the different scaling methods, the parameters *p_g_* were also scaled based on silhouette area (*a*_sil_), *w*, and age (*a*). The scaling method was performed in the same way as in Equation 7, by simply replacing silhouette length with silhouette area, body weight, or age. This yields other variations of the scaled gait parameters ${\hat{p}}_{g}$, as follows:

(9) $${\hat{p}}_{g}=\frac{p_{g}}{a_{sil}}$$

(10) $${\hat{p}}_{g}=\frac{p_{g}}{\text{w}}$$

(11) $${\hat{p}}_{g}=\frac{p_{g}}{a}$$

### Code accessibility

The computer code for calculating the silhouette length (see Silhouette length and silhouette area computation) together with data from several examples are freely available on-line at https://github.com/mad-lab-fau/SilhouetteLength.

### Analysis

Silhouette length values were computed for all runs or videos of each animal in accordance with the description in the section Silhouette length and silhouette area computation. The silhouette length, gait parameters, as well as the scaled gait parameters computed from all run videos were subsequently averaged for each animal. Gait parameter scaling was performed for all the parameters listed in the section Study protocol and data acquisition. Scaled parameters were calculated for each run and averaged for each animal. Sample Pearson correlation coefficient (Pearson, 1895) *r*, coefficient of determination *r* ^2^, and the *p*- value between the silhouette length and gait parameters were computed. The *r* and *p* values before and after scaling were compared. For all gait parameters showing higher *p* values (and lower *r* values) on their scaled version, a repeated-measures ANOVA was performed based on animals that were tested consecutively at all age points. This repeated-measures ANOVA was also performed on the silhouette parameters (length and area) to investigate age-related effects.

The computed silhouette length values were compared with the physically measured body length values. This comparison was performed in the small cohort of rats by observing the correlation and the difference between silhouette length and body length.

The comparison study among silhouette-length-, silhouette-area-, weight-, and age-based scaling was performed in accordance with the scaling method in Equation 7, by simply replacing silhouette length with silhouette area, body weight, or age, as shown in Equations 9–11. Then, we evaluated both the *r* and *p* values of the correlation between gait parameters and silhouette area/weight/age. The body-weight-based scaling was performed by using the animals with corresponding body weight information.

Genotype-related differences were investigated via mixed ANOVA (occasionally also called two-way repeated-measures ANOVA) and multiple comparison tests with Bonferroni correction in Matlab R2015a (version 8.5.0.). The *p* values with lower bound adjustments were used to detect the presence of significant differences and were reported by using heat maps (Timotius et al., 2018a, 2019).

## Results

### Silhouette length and silhouette-length-based gait parameter scaling in wild-type rodents

**Figure 4.**
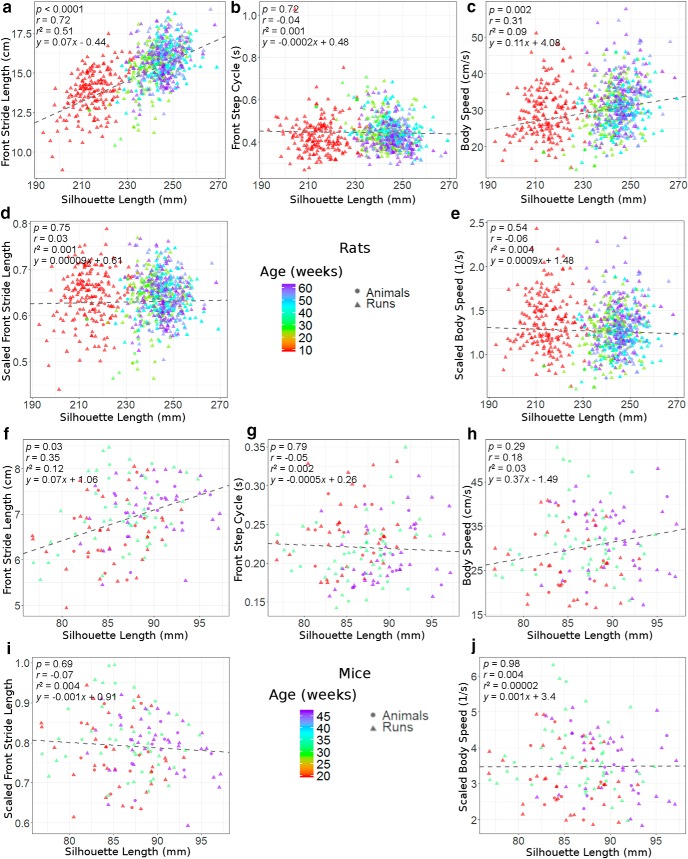
Scatter plots for the correlation between nonscaled and scaled gait parameters with silhouette length in wild-type rodents. The numbers of animals and the number of run videos are given in Figure 1. The animal data are the averaging results from their respective CatWalk run data. *r* values, *r* ^2^ values, *p* values, and the regression line were calculated from the animal data.

The scatter plots of several gait parameters as a function of silhouette length for rats and mice are depicted in Figure 4. The color in the scatter plots denotes the age of the animals, with young rodents showing smaller silhouette lengths compared with older ones. The correlation coefficients between the gait parameters and silhouette length are given in Table 1. No significant correlation with the silhouette length was shown for the gait parameters relating to duration. Significant correlations (*p* < 0.05) were observed between stride length and silhouette length in rats as well as mice. Significant correlations in rats were also shown between the parameters related to speed and silhouette length, whereas only moderate correlations were shown in mice.

**Table 1.** Sample Pearson correlation coefficients (*r*) and *p* values of the silhouette length and CatWalk gait parameters

	Rats				Mice			
Gait	Nonscaled		Scaled		Nonscaled		Scaled	
parameters	*r*	*p*	*r*	*p*	*r*	*p*	*r*	*p*
Stride lengths								
Front-paw stride length	0.72	<0.0001	**0.03**	**0.75**	0.35	0.03	**−0.07**	**0.69**
Hind-paw stride length	0.72	<0.0001	**0.02**	**0.81**	0.35	0.03	**−0.05**	**0.76**
Parameters relating to duration								
Front-paw stand time	0.02	0.81	−0.34	<0.001	<0.01	0.97	−0.18	0.28
Front-paw swing time	−0.20	0.05	−0.58	<0.0001	−0.12	0.48	−0.38	0.02
Front-paw step cycle	−0.04	0.72	−0.45	<0.0001	−0.05	0.79	−0.27	0.10
Hind-paw stand time	−0.05	0.59	−0.36	<0.001	<0.01	0.97	−0.16	0.33
Hind-paw swing time	0.07	0.51	−0.57	<0.0001	−0.10	0.56	−0.42	<0.01
Hind-paw step cycle	−0.04	0.70	−0.46	<0.0001	−0.03	0.85	−0.26	0.12
Speed parameters								
Body speed	0.31	<0.01	**(i) −0.06** **(ii) 0.13**	**(i) 0.54** **(ii) 0.20**	0.18	0.29	**(i)<0.01** **(ii) 0.09**	**(i) 0.98** **(ii) 0.58**
Front-paw swing speed	0.49	<0.0001	**(i) 0.09** **(ii) 0.31**	**(i) 0.40** **(ii)<0.01**	0.22	0.18	**(i) 0.02** **(ii) 0.12**	**(i) 0.90** **(ii) 0.46**
Hind-paw swing speed	0.51	<0.0001	**(i) −0.08** **(ii) 0.25**	**(i) 0.43** **(ii) 0.01**	0.30	0.07	**(i) 0.04** **(ii) 0.18**	**(i) 0.80** **(ii) 0.29**

The scaled parameters which show higher *p* values compared with their nonscaled versions are in bold. (i), Scaling method: ${\hat{p}}_{g}=p_{g}/l_{sil}$; (ii), scaling method: ${\hat{p}}_{a}=p_{g}/\sqrt{g/l_{sil}}$.

The scaling approaches were performed for all parameters listed in the section Study protocol and data acquisition. For the stride lengths and the parameters relating to speed, the scaled parameters showed lower *r* values (and higher *p* values) compared with nonscaled parameters. The speed parameters, which were scaled using Equation 7, displayed lower correlation coefficients compared with the parameters scaled using Equation 8.

**Figure 5.**
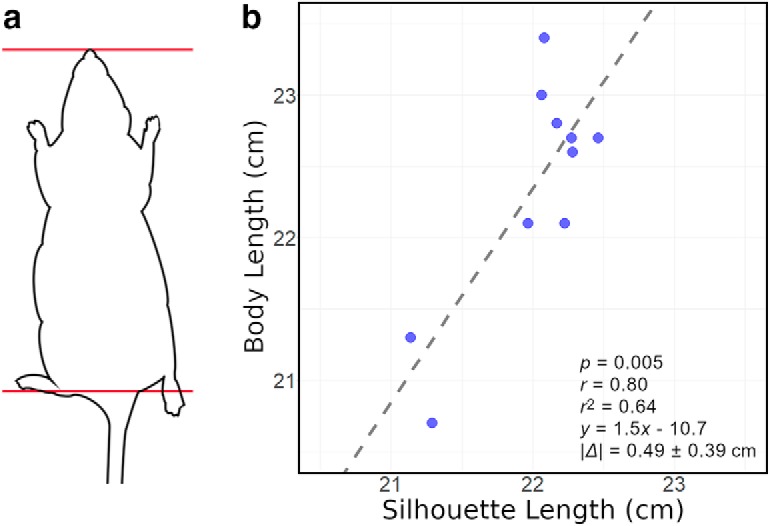
***a***, Diagram of body length (nose-tip to tail-base) measurement. ***b***, Scatter plot for correlation between body length and silhouette length of the small cohort of rats.

The relation between silhouette length and measured body length calculated from the small cohort of rats is depicted in Figure 5*b*. The body length was measured from the nose-tip to tail-base, as shown in Figure 5*a*. A significant correlation between silhouette length and body length was shown (*p* < 0.01). The mean ± SD of the absolute difference between silhouette length and body length |Δ| was 0.49 ± 0.39 cm.

The age analysis of wild-type animals using repeated-measure ANOVA is given in Table 2. This analysis was conducted for the gait parameters that show significant correlations in Table 1 and for the silhouette parameters. In rats, significant effects of age are shown for silhouette parameters and gait parameters, excluding the body speed. In mice, a significant effect of age was shown for silhouette parameters and swing speeds. All of the scaled gait parameters listed in Table 2 show higher *p* values compared with their corresponding nonscaled parameters.

**Table 2.** The effect of age on the silhouette and gait parameters: repeated-measures ANOVA (*p* value**s)**

	Rats		Mice	
Parameters	Nonscaled	Scaled*	Nonscaled	Scaled*
Stride lengths				
Front-paw stride length	<0.001	0.69	0.13	0.20
Hind-paw stride length	<0.0001	0.64	0.12	0.18
Speed parameters				
Body speed	0.11	0.24	0.11	0.15
Front-paw swing speed	0.03	0.35	0.04	0.07
Hind-paw swing speed	0.04	0.24	0.04	0.06
Silhouette parameters				
Silhouette length	<0.0001		<0.01	
Silhouette area without tail	<0.0001		<0.001	
Silhouette area with tail	<0.0001		<0.001	

*Scaling method ${\hat{p}}_{g}=p_{g}/l_{sil}$.

### Silhouette area-, body-weight-, and age-based gait parameter scaling in wild-type rodents

**Figure 6.**
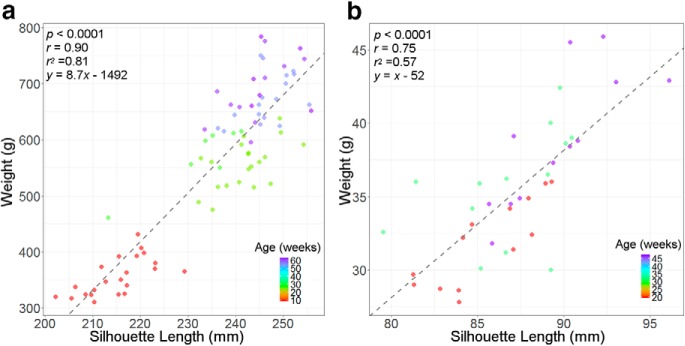
***a***, ***b***, Scatter plots for correlation between body weight with silhouette length of wild-type animals: rats (***a***) and mice (***b***).

As rodents grow, it is reasonable to expect that the body silhouette length, body silhouette area, and body weight also increase. Correspondingly, high correlation among them, as well as age, were found (Table 3). The relationship between body weight and silhouette length for rats and mice is also reported as the scatter plots shown in Figure 6.

**Table 3.** Correlation between silhouette length, silhouette area (with tail), body weight, and age: *p* values, *r* values, and *r* ^2^ values

	Silhouette length		Silhouette area		Body weight			Age	
	Rats	Mice	Rats	Mice	Rats	Mice		Rats	Mice
Silhouette length	*p* =0 *r* = 1 *r^2^* = 1								
Silhouette area	*p* < 0.0001 *r* = 0.93 *r^2^* = 0.87	*p* < 0.0001 *r* = 0.84 *r^2^* = 0.70	*p* = 0 *r* = 1 *r^2^* = 1						
Body weight	*p* < 0.0001 *r* = 0.90 *r^2^* = 0.81	*p* < 0.0001 *r* = 0.75 *r^2^* = 0.57	*p* < 0.0001 *r* = 0.98 *r^2^* = 0.96	*p* < 0.0001 *r* = 0.89 *r^2^* = 0.79	*p* = 0 *r* = 1 *r^2^* = 1				
Age	*p* < 0.0001 *r* = 0.74 *r^2^* = 0.55	*p* < 0.01 *r* = 0.49 *r^2^* = 0.24	*p* < 0.0001 *r* = 0.88 *r^2^* = 0.77	*p* < 0.01 *r* = 0.50 *r^2^* = 0.25	*p* < 0.0001 *r* = 0.90 *r^2^* = 0.81		*p* < 0.0001 *r* = 0.61 *r^2^* = 0.37	*p* = 0 *r* = 1 *r^2^* = 1	

The correlation coefficients between the silhouette area (*a*_sil_) and the CatWalk gait parameters are given in Table 4. The parameter scaling in Table 4 was performed based on the silhouette area. In rats, significant correlations (*p* < 0.05) with the silhouette area were shown for the gait parameters relating to stride length or speed, but not for the parameters relating to duration. All silhouette-area-based scaled gait parameters showed significant correlations with the silhouette area. In mice, most of the silhouette-area-scaled gait parameters gave lower *p* values compared with the nonscaled parameters. Some exceptions were revealed for the stand-time parameters, where both of the nonscaled and scaled gait parameters showed no significant correlation with the silhouette area.

**Table 4.** *r* and *p* values of the silhouette area (*a*_sil_) and CatWalk gait parameters (scaling method: ${\hat{p}}_{g}=p_{g}/a_{sil}$**)**

	Rats				Mice			
Gait	Nonscaled		Scaled		Nonscaled		Scaled	
parameters	*r*	*p*	*r*	*p*	*r*	*p*	*r*	*p*
Stride lengths								
Front-paw stride length	0.59	<0.0001	−0.80	<0.0001	0.31	0.06	−0.37	0.02
Hind-paw stride length	0.59	<0.0001	−0.81	<0.0001	0.31	0.06	−0.35	0.03
Parameters relating to duration								
Front-paw stand time	0.06	0.57	−0.66	<0.0001	0.19	0.25	**−0.14**	**0.41**
Front-paw swing time	−0.14	0.17	−0.80	<0.0001	<0.001	0.99	−0.45	<0.01
Front-paw step cycle	0.01	0.88	−0.74	<0.0001	0.11	0.49	−0.29	0.08
Hind-paw stand time	<0.01	0.94	−0.62	<0.0001	0.15	0.37	**−0.13**	**0.43**
Hind-paw swing time	0.02	0.81	−0.88	<0.0001	0.06	0.71	−0.49	<0.01
Hind-paw step cycle	<0.01	0.99	−0.76	<0.0001	0.13	0.44	−0.27	0.10
Speed parameters								
Body speed	0.27	0.02	−0.55	<0.0001	0.03	0.85	−0.24	0.15
Front-paw swing speed	0.38	<0.0001	−0.54	<0.0001	0.13	0.45	−0.20	0.22
Hind-paw swing speed	0.44	<0.0001	−0.72	<0.0001	0.15	0.36	−0.26	0.11

The scaled parameters that show higher *p* values compared with their nonscaled versions are in bold.

The correlation coefficients between *w* and the CatWalk gait parameters are given in Table 5. The scaling method in Table 5 was based on body weight. Both in rats and mice, significant correlations were found between the body weight and body-weight-scaled gait parameters.

**Table 5.** *r* and *p* values of *w* and CatWalk gait parameters (scaling method: ${\hat{p}}_{g}=p_{g}/w$**)**

	Rats				Mice			
Gait	Nonscaled		Scaled		Nonscaled		Scaled	
parameters	*r*	*p*	*r*	*p*	*r*	*p*	*r*	*p*
Stride lengths								
Front-paw stride length	0.54	<0.0001	−0.95	<0.0001	0.36	0.03	−0.69	<0.0001
Hind-paw stride length	0.54	<0.0001	−0.95	<0.0001	0.37	0.02	−0.67	<0.0001
Parameters relating to duration								
Front-paw stand time	0.04	0.70	−0.87	<0.0001	0.06	0.73	−0.47	<0.01
Front-paw swing time	−0.11	0.35	−0.92	<0.0001	−0.01	0.95	−0.67	<0.0001
Front-paw step cycle	0.01	0.92	−0.90	<0.0001	0.02	0.88	−0.58	<0.001
Hind-paw stand time	0.01	0.91	−0.84	<0.0001	0.04	0.81	−0.42	<0.01
Hind-paw swing time	−0.01	0.90	−0.95	<0.0001	0.02	0.88	−0.74	<0.0001
Hind-paw step cycle	<0.01	0.98	−0.91	<0.0001	0.03	0.86	−0.57	<0.001
Speed parameters								
Body speed	0.20	0.08	−0.81	<0.0001	0.10	0.54	−0.39	0.02
Front-paw swing speed	0.32	<0.01	−0.83	<0.0001	0.14	0.39	−0.43	<0.01
Hind-paw swing speed	0.41	<0.001	−0.90	<0.0001	0.21	0.21	−0.50	<0.01

The correlation coefficients and *p* values between age and the CatWalk gait parameters are given in Table 6. Significant correlations were found between the age and age-scaled gait parameters.

**Table 6.** *r* and *p* values of the age (*a*) and CatWalk gait parameters (scaling method: ${\hat{p}}_{g}=p_{g}/a$**)**

	Rats				Mice			
Gait	Nonscaled		Scaled		Nonscaled		Scaled	
parameters	*r*	*p*	*r*	*p*	*r*	*p*	*r*	*p*
Stride lengths								
Front-paw stride length	0.55	<0.0001	−0.89	<0.0001	0.33	0.046	−0.93	<0.0001
Hind-paw stride length	0.57	<0.0001	−0.89	<0.0001	0.33	0.044	−0.93	<0.0001
Parameters relating to duration								
Front-paw stand time	−0.05	0.62	−0.88	<0.0001	−0.24	0.14	−0.84	<0.0001
Front-paw swing time	−0.14	0.16	−0.88	<0.0001	−0.33	0.04	−0.91	<0.0001
Front-paw step cycle	−0.07	0.50	−0.89	<0.0001	−0.31	0.06	−0.89	<0.0001
Hind-paw stand time	−0.08	0.44	−0.88	<0.0001	−0.21	0.20	−0.81	<0.0001
Hind-paw swing time	0.08	0.41	−0.89	<0.0001	−0.35	0.03	−0.93	<0.0001
Hind-paw step cycle	−0.08	0.45	−0.89	<0.0001	−0.29	0.07	−0.88	<0.0001
Speed parameters								
Body speed	0.28	<0.01	−0.85	<0.0001	0.32	0.047	−0.74	<0.0001
Front-paw swing speed	0.36	<0.001	−0.86	<0.0001	0.38	0.02	−0.81	<0.0001
Hind-paw swing speed	0.37	<0.001	−0.88	<0.0001	0.44	<0.01	−0.87	<0.0001

### Genotype-related differences in silhouette-length-based scaled gait parameters in rodent models of neurodegenerative diseases

**Figure 7.**
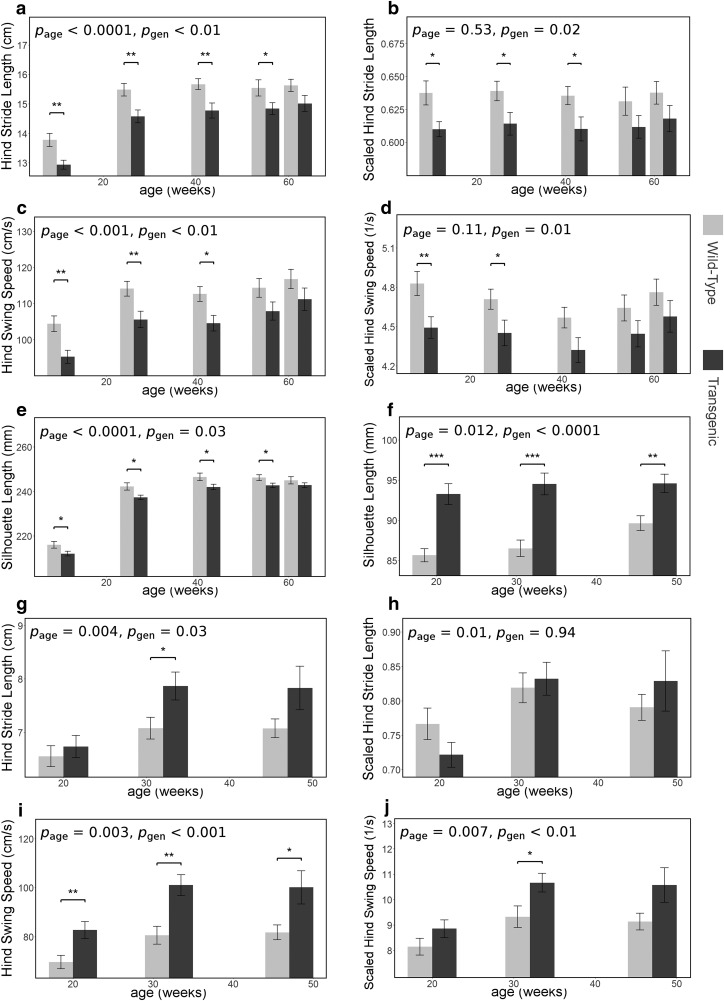
***a–j***, Genotype-related differences in unscaled and scaled hind stride length, unscaled and scaled hind swing speed, as well as silhouette length along with age: α-synuclein rat (***a–e***), BACHD mice (***f–j***). Each data point represents the mean ± SEM. *p* Values from multiple-comparison tests with Bonferroni correction: *p < 0.05, **p < 0.01, ***p < 0.001.

**Figure 8.**
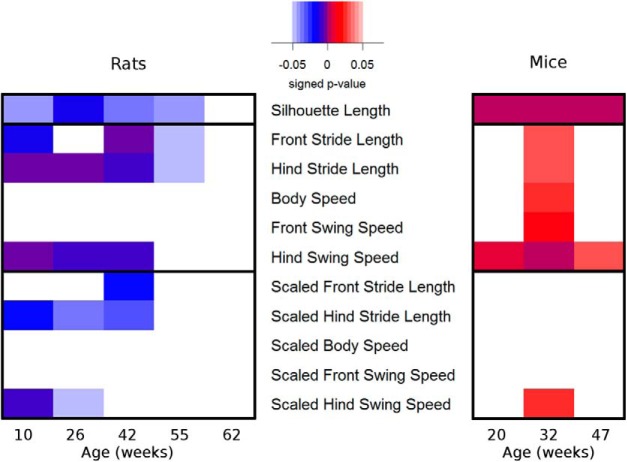
Differences between wild-type and transgenic animal models observed by their unscaled and scaled gait parameters individually by multiple-comparison tests and reported in heat maps (Timotius et al., 2018a, 2019).

As a proof of concept for our scaling approach, we examined genotype-related differences for BAC α-Syn transgenic and BACHD animals as well as the effect of age and their interaction, as shown in Table 7. The mean and SEM of the silhouette lengths and several gait parameters are depicted in Figure 7. In addition, the *p* values of the multiple comparison tests are depicted by heat maps (Timotius et al., 2018a, 2019) in Figure 8. The color blue in the heat maps represents lower parameter value of the transgenic rodents compared with their corresponding wild type, whereas the color red in the heat maps represents the higher-parameter values of the transgenic rodents compared with their corresponding wild types.

**Table 7.** The effect of age, the genotype–age interaction and the effect of genotype on the silhouette parameters and gait parameters: mixed ANOVA and multiple-comparison tests with Bonferroni correction (*p* values with lower bound adjustment)

	Rats			Mice		
Parameters	Age	Interaction	Genotype	Age	Interaction	Genotype
Stride lengths						
Front-paw stride length (cm)	<0.0001	0.46	0.01	0.005	0.25	0.03
Hind-paw stride length (cm)	<0.0001	0.54	<0.01	0.004	0.24	0.03
Scaled front-paw stride length^*^	0.60	0.58	0.046	0.013	0.17	0.90
Scaled hind-paw stride length^*^	0.53	0.67	0.02	0.01	0.17	0.94
Speed parameters						
Body speed (cm/s)	0.01	0.55	0.26	0.004	0.25	0.03
Front-paw swing speed (cm/s)	<0.001	0.43	0.35	0.004	0.43	<0.01
Hind-paw swing speed (cm/s)	<0.001	0.59	<0.01	0.003	0.47	<0.001
Scaled body speed (1/s)^*^	0.16	0.51	0.47	0.007	0.25	0.18
Scaled front-paw swing speed (1/s)^*^	0.13	0.41	0.65	0.007	0.45	0.11
Scaled hind-paw swing speed (1/s)^*^	0.11	0.60	0.01	0.007	0.47	<0.01
Body parameters						
Silhouette length (mm)	<0.0001	0.23	0.03	0.012	0.10	<0.0001
Silhouette area without tail (mm^2^)	<0.0001	0.15	<0.001	<0.001	0.26	<0.0001
Silhouette area with tail (mm^2^)	<0.0001	0.26	<0.01	<0.01	0.14	<0.0001
Weight (g)	<0.0001	0.20	0.03	<0.0001	0.35	<0.0001
Weight silhouette length index (g/cm^2^)	<0.0001	0.17	0.29	<0.001	0.55	<0.001

* Scaled based on the silhouette length (scaling method: ${\hat{p}}_{g}=p_{g}/l_{sil}$).

As shown in Table 7, all nonscaled gait parameters (stride lengths and speed-related gait parameters) and silhouette parameters showed significant effects of age (*p* < 0.05). In rats, the silhouette-length-based scaled gait parameters did not show any significant effect of age, whereas in mice, reduced age effects were observed in scaled gait parameters. No interaction between age and genotype in any gait parameter was observed. The genotype effects shown by the scaled gait parameters were lower compared with their corresponding nonscaled gait parameters. Besides the silhouette parameters, significant differences between genotypes were observed in scaled front stride length, scaled hind stride length, and scaled hind swing speed.

Significant genotype-related differences in the scaled front stride length were observed in 42-week-old rats. The scaled hind stride length showed significant differences between genotypes in the young rats until they were 42 weeks old. Furthermore, the scaled hind swing speed showed significant genotype-related differences for 10- and 26-week-old rats. In mice, significant differences of scaled gait parameters are shown only for scaled hind swing speed at 32 weeks of age.

## Discussion

As in humans, several rodent gait parameters are correlated with body size. This study presents a body silhouette length computation method for rodents based on the recorded CatWalk videos. A correlational study was performed to investigate the relationship between the computed silhouette length and several CatWalk gait parameters. Accordingly, a silhouette-length-based scaling method on stride lengths and speed-related gait parameters is presented, which is able to reduce their correlation with body size. This silhouette-length-based scaling method cannot be directly replaced by silhouette-area-, body-weight-, or age-based scaling methods. As proof of concept, two rodent models of neurodegenerative disorders were investigated based on their scaled gait parameters. The results show smaller genotype-related differences compared with the nonscaled gait parameters due to the genotype-related body size difference.

The computed silhouette parameters, stride lengths, and speed-related gait parameters were found to change with advancing age. Significant effects of age on silhouette length and silhouette area, both with and without tail area, were observed in both rats and mice. Correlations between body silhouette length and stride lengths were significant for both rats and mice. The silhouette-length-based scaling/normalization reduced this correlation. The effect of age on stride lengths was significant in rats, but not in mice. However, in both species, the scaled parameters gave higher *p* values in the repeated-measures ANOVA compared with the nonscaled parameters. The effect of age on silhouette and gait parameters, as well as the effect of scaling on gait parameters, was more visible in rats compared with mice since the age range of the rats was broader than that of the mice. There was no significant effect of age on the scaled stride length. This suggests that the scaled stride lengths were better tools for a longitudinal study compared with the nonscaled stride lengths.

Significant correlations between silhouette length and speed (or velocity) parameters were observed in rats, but not in mice. Nevertheless, in rats and mice, both scaling methods gave scaled speed parameters with higher *p* values compared with the nonscaled version. The first scaling method, in accordance with Equation 7, was more suitable for the scaling of speed-related gait parameters. As speed is proportional to length and is inversely proportional to time, along with considering the nonsignificant correlation of duration-related gait parameter to the silhouette length, it is reasonable that the scaling method of the length-related gait parameters was also suitable for the speed-related gait parameters. Walking speed is a gait parameter that is affected by many factors (Batka et al., 2014). Therefore, it is reasonable that the correlations of silhouette length to the speed-related gait parameters were not as strong as the correlations to the length-related gait parameters.

No significant correlation was found in the gait parameters relating to duration. The silhouette length scaling on duration-related gait parameters produces scaled gait parameters, which are more correlated with silhouette length compared with the nonscaled version. This suggests that no silhouette-length-based scaling is needed for duration-related gait parameters.

In humans, gait parameters are typically evaluated by presenting data in the form of nondimensional numbers (Hof, 1996; Zijlstra et al., 1996). This scaling method suggested for human gait is not relevant to the practice in rodents. We recommend silhouette-length-based scaling in the stride lengths and speed parameters of rodents, but not in duration-related gait parameters since the duration-related gait parameters in rodents do not significantly correlate with silhouette-based body length.

The measured body lengths of anesthetized rats show significant correlation with the silhouette lengths. The measured body lengths were similar to the silhouette lengths with an absolute mean ± SD difference of 0.49 ± 0.39 cm. Most of the measured body lengths are higher compared with the silhouette length. This difference occurs most likely because of the different body curve while lying on a plain surface versus walking.

Since silhouette length, silhouette area, and body weight are all related to body size, they were all correlated to each other. However, replacing silhouette length with silhouette area for scaling is not recommended since the silhouette-area-based-scaled stride lengths and speed parameters were significantly correlated with the silhouette area. The same can be also noticed in the body-weight-based gait parameter scaling. The duration-related gait parameters did not display significant correlation with silhouette length or with body weight.

Although body size changes with the advancing age of the animal, silhouette-length-based gait parameter scaling should not be replaced by age-based parameter scaling. This is due to the fact that the age-based scaled gait parameters have significant correlations with age, which in all likelihood happened because the animals grow faster early in their lives (Lui and Baron, 2011). Therefore, age and body size do not have a linear correlation.

As rodents do not grow linearly (Lui and Baron, 2011), wild-type rats in our study grew over the months and reached the greatest silhouette length by the age of 10 months. Afterward, their silhouette length decreased slightly, possibly due to postural changes (hunched back). The wild-type mice showed an increase in silhouette length with advancing age.

In animal models with genotype-related body size differences, gait parameters can be directly and indirectly (through body size) related to the genotype. In the analysis of genotype-related differences as described in the section Genotype-related differences in silhouette-length-based scaled gait parameters in rodent models of neurodegenerative diseases, the effect of age was observed on all body parameters and nonscaled gait parameters. The genotype and age of the animals had lower effects on the scaled gait parameters compared with the nonscaled gait parameters for rodent models with genotype-related body size differences. No significant genotype–age interaction was observed. Considering the results shown in Figures 7 and 8, significant genotype-driven silhouette length differences were observed. Additionally, fewer genotype-related gait parameter differences were detected by the scaled gait parameters. Gait analysis in preclinical studies is mostly aimed at the development of new treatment strategies for specific diseases. Observing the effects of diseases and treatments on gait is often more important than observing the effect of body size on gait. Therefore, gait parameters used in the analysis of gait should have a minimal relation to body size. Accordingly, the results in Figures 7 and 8 yield an analytic value improvement, as the scaling/normalizing approach reduces the effect of body size. Therefore, it can be confirmed that gait parameter scaling is recommended in a study involving rodent models with significant genotype-related silhouette length differences.

Significant genotype-related differences in the α-synuclein rat model was detected on scaled stride lengths, scaled hind-paw swing speed, silhouette length, silhouette area, and body weight. By observing the silhouette length, area, and body weight, the α-synuclein rats were smaller compared with their wild-type littermates. Genotype-related differences in the α-synuclein rat model were more pronounced in young animals than in geriatric animals.

In the BACHD mouse model, a genotype-related difference was observed on scaled hind-paw swing speed, specifically in 32-week-old mice. The body parameters show that the BACHD mice are bigger compared with their wild-type littermates.

Further possible applications of the line representing the silhouette length are in the development of a 2D Eshkol Wachmann Notation (Eilam and Golani, 1988) and body coordinate frame (Neckel, 2015). As body silhouettes provided by the CatWalk system are the projection of the body of the rodent, body silhouette length is not the same as body length. A further comparison study based on body length (mouth–anus) could offer insight into its usage for gait parameter scaling, despite the fact that anesthesia would be needed. Since body weight is correlated with print dimensions (print length, width, and area; Zimprich et al., 2018), a further study on print dimension parameters scaling by body weight is recommended. Furthermore, a correlation study between the print position (distance between the position of the hindpaw and the position of the previously placed front paw on the same side of the body) and body size might also be valuable for gait analysis in rodents. Moreover, a similar study on the relation between body size and treadmill-based gait parameters (Sparrow et al., 2017) will be valuable for the field of preclinical gait analysis.

In summary, a method of calculating body silhouette length is described in this article. This silhouette length is beneficial for the scaling of stride length and speed-related gait parameters. Gait parameter scaling is suggested to be an important tool for augmenting the reliability of motor function analyses in rodent models, especially in studies involving young animals and/or rodent models with genotype-related silhouette length differences, as well as in longitudinal studies.

## Synthesis

Reviewing Editor: Tatyana Sharpee, The Salk Institute for Biological Studies Decisions are customarily a result of the Reviewing Editor and the peer reviewers coming together and discussing their recommendations until a consensus is reached. When revisions are invited, a fact-based synthesis statement explaining their decision and outlining what is needed to prepare a revision will be listed below. The following reviewer(s) agreed to reveal their identity: NONE.

In the manuscript titled “Silhouette-length-scaled gait parameters for motor functional analysis in mice and rats,” the authors attempt to address the issue that variation in body size can confound automated gait tracking analysis. Using silhouette-derived body length parameters, as well as silhouette area, body weight, and age, they test which scaling approaches most appropriately dissociate kinematic parameters from body size, finding that normalizing for silhouette length produces the best decorrelation. They then apply this approach to analysis of rodent motor disease models, demonstrating that genotype-related differences in body size affect the assessment of locomotor kinematics, including stride length, body speed, and swing speed.

Overall, this manuscript provides convincing evidence that scaling for silhouette length reduces correlations with body size that can affect kinematic analysis. Moreover, that fact that silhouette length can be measured from the same locomotion dataset (rather than through independent measurement in anesthetized rodents, for example) provides a convenient and efficient way to achieve the scaling they describe. That said, some major assumptions are made that need to be explained and justified a bit more clearly.

1. It seems that the major goal of this manuscript is to achieve a more reliable measure of the direct relationship between genotype-to-gait (direct effect), without the potential confound of an indirect relationship between genotype-to-body size-to-gait (indirect effect). It would be helpful if the authors explicitly describe this framework of direct and indirect causality and how they both affect gait. There is an assumption that the direct and indirect effects are completely independent, and normalizing one will not affect the other. Why does genotype influence on gait through body size (indirect effect) inherently confound gait analysis? It does seem like a reasonable goal to dissociate direct and indirect effects of genotype on gait, but this goal and the assumptions behind it were never clearly described or justified.

An example of an assumption that needs to be more clearly explained is that the indirect effect of genotype on gait (through body length) has multiplicative confounding effects, and therefore dividing body length out of the gait parameters removes the confound. Again, this might be correct, but the logical arguments behind their assumptions are never explained.

Another example is that on lines 405-408, the authors recommend normalizing only those gait parameters that exhibit a significant correlation with silhouette length. This argument lacks a convincing explanation. It seems that the authors argue that if something is correlated with body length, it is inherently confounding. While this might be a reasonable assumption, the authors should discuss their causal logic more explicitly, including any caveats to their assumptions.

2. The manuscript lacks comparison with physical measurements (ground truth) to validate their technology. It would be nice to see some physical confirmation of the computed silhouette length (i.e. take a physical measurement of body length on a subset of anesthetized animals and compare to the computed values).

Minor point: check for awkward wording and typos throughout- e.g. line 90 “as very often desirable”, line 715 “of a function with age”, etc.

## Author Response

The authors of the manuscript eNeuro eN-MNT-0100-19 entitled “Silhouette-length-scaled gait parameters for motor functional analysis in mice and rats” would like to thank the reviewers for the valuable and insightful comments for the manuscript. The present version of our manuscript has been carefully revised following the comments in every regard. All changes are marked in our revised manuscript. A point-by-point reply to all specific issues and comments follows.

Computational Neuroscience Model Code Accessibility Comments for Author (Required):

There is no separate “code accessibility” section but the data collection procedure was clearly described. The authors used the Catwalk system for markerless gait analysis (Hamers et al., 2006, 136 2001; Noldus Information Technology b.v., 2012).

Response:

Code accessibility section is added to the manuscript (Section 2.5).

Significance Statement Comments for Author (Required):

The statement is well written

Response:

-

Comments on the Visual Abstract for Author (Required):

The abstract is informative and conveys the major conclusion

Response:

-

Synthesis Statement for Author (Required):

In the manuscript titled “Silhouette-length-scaled gait parameters for motor functional analysis in mice and rats,” the authors attempt to address the issue that variation in body size can confound automated gait tracking analysis. Using silhouette-derived body length parameters, as well as silhouette area, body weight, and age, they test which scaling approaches most appropriately dissociate kinematic parameters from body size, finding that normalizing for silhouette length produces the best decorrelation. They then apply this approach to analysis of rodent motor disease models, demonstrating that genotype-related differences in body size affect the assessment of locomotor kinematics, including stride length, body speed, and swing speed.

Overall, this manuscript provides convincing evidence that scaling for silhouette length reduces correlations with body size that can affect kinematic analysis. Moreover, that fact that silhouette length can be measured from the same locomotion dataset (rather than through independent measurement in anesthetized rodents, for example) provides a convenient and efficient way to achieve the scaling they describe. That said, some major assumptions are made that need to be explained and justified a bit more clearly.

Point 1a:

1. It seems that the major goal of this manuscript is to achieve a more reliable measure of the direct relationship between genotype-to-gait (direct effect), without the potential confound of an indirect relationship between genotype-to-body size-to-gait (indirect effect). It would be helpful if the authors explicitly describe this framework of direct and indirect causality and how they both affect gait. There is an assumption that the direct and indirect effects are completely independent, and normalizing one will not affect the other. Why does genotype influence on gait through body size (indirect effect) inherently confound gait analysis? It does seem like a reasonable goal to dissociate direct and indirect effects of genotype on gait, but this goal and the assumptions behind it were never clearly described or justified.

Response:

The description of the framework of direct (genotype-to-gait) and indirect (genotype-to-body_size-to-gait) causality was added in Section 4 Paragraph 9.

Normalizing the body_size-to-gait effect will reduce the effect of genotype-to-body_size-to-gait, which makes it possible for researchers to focus more on the genotype-to-gait effect.

Gait analysis in preclinical studies is mostly aimed at the development of new treatment strategies for specific diseases. Observing the effect of diseases and treatments on gait is often more important than observing the effect of body size on gait. Therefore, gait parameters used in the analysis of gait should have minimal relation with body size. This goal was added in Section 4 Paragraph 9.

Point 1b:

An example of an assumption that needs to be more clearly explained is that the indirect effect of genotype on gait (through body length) has multiplicative confounding effects, and therefore dividing body length out of the gait parameters removes the confound. Again, this might be correct, but the logical arguments behind their assumptions are never explained.

Response:

We would like to acknowledge the reviewers for pointing out the inequality between correlation and causality. We agree with the reviewers that we should not assume: correlation always imply direct causality. The manuscript is therefore revised to the direction of correlation study, as here (described in Section 3.1) we present that: several gait parameters are related with body length and dividing it with the body length can reduce the relationship.

Point 1c:

Another example is that on lines 405-408, the authors recommend normalizing only those gait parameters that exhibit a significant correlation with silhouette length. This argument lacks a convincing explanation. It seems that the authors argue that if something is correlated with body length, it is inherently confounding. While this might be a reasonable assumption, the authors should discuss their causal logic more explicitly, including any caveats to their assumptions.

Response:

We would like to again thanks the reviewers for pointing out that correlation do not imply causality. The description of causality and how body-size affect gait in rodents will need an elaboration of body mechanism and muscle activity, in which a broader study design is needed. Therefore, we revised the manuscript emphasizing more to the correlation/relation between gait parameters and body size.

Point 2:

2. The manuscript lacks comparison with physical measurements (ground truth) to validate their technology. It would be nice to see some physical confirmation of the computed silhouette length (i.e. take a physical measurement of body length on a subset of anesthetized animals and compare to the computed values).

Response:

Additional study comparing silhouette length and body length based on physical measurement is presented in Section 3.1.

Point 3:

Minor point: check for awkward wording and typos throughout- e.g. line 90 “as very often desirable”, line 715 “of a function with age”, etc.

Response:

Thank you for pointing this out. The sentences has been changed.
